# Clonal Multidrug-Resistant *Corynebacterium striatum* Strains, Italy

**DOI:** 10.3201/eid1501.080804

**Published:** 2009-01

**Authors:** Floriana Campanile, Edoardo Carretto, Daniela Barbarini, Annalisa Grigis, Marco Falcone, Antonio Goglio, Mario Venditti, Stefania Stefani

**Affiliations:** University of Catania, Catania, Italy (F. Campanile, S. Stefani); Policlinico “San Matteo,” Pavia, Italy (E. Carretto, D. Barbarini); Ospedali Riuniti, Bergamo, Italy (A. Grigis, A. Goglio); University Roma La Sapienza, Rome, Italy (M. Falcone, M. Venditti)

**Keywords:** Corynebacterium striatum, multi-resistance plasmid pTP10, emerging clinical role, dispatch

## Abstract

We assessed the clinical relevance and performed molecular characterization of 36 multidrug-resistant strains of *Corynebacterium striatum.* Pulsed-field gel electrophoresis confirmed a single clone, possessing *erm*(X), *tet*A/B, *cmx*A/B, and *aph*A1 genes, but few related subclones. This strain is emerging as a pathogen in Italy.

Isolation of *Corynebacterium* spp. as the only organism from clinical specimens from patients, mostly with varying degrees of immunocompromisation and severe infections, is increasing in Italy. Therefore, we evaluated the microbiologic characteristics, resistance profiles, and similarities among genomes of multidrug-resistant (MDR) *C. striatum* strains.

## The Study

We evaluated 36 strains of MDR *C. striatum,* isolated from 3 hospitals in Italy during 2005–2007. Fourteen strains were from bronchoalveolar lavage (BAL) fluid, 3 from blood, 7 from central venous catheter tips, 5 from tracheal aspirates, 4 from wound specimens, 1 from BAL and pleural fluid, 1 from urine, and 1 from a lung biopsy specimen. To assess the clinical relevance of these strains, we used the Centers for Disease Control and Prevention 2004 definition for nosocomial infections (www.cdc.gov/ncidod/dhqp/nnis_pubs.html) (*1*) and tracked antimicrobial drug–resistance determinants.

We identified all strains as putative *C. striatum* by using the commercial system API 20 Coryne (bioMérieux, Marcy l’Etoile, France). *C. striatum* was differentiated from *C. amycolatum* by supplementary tests, i.e., tyrosine hydrolysis, N-acetylglucosamine assimilation, and phenylacetic acid assimilation (*2*); it was reconfirmed by sequencing the internal fragment of the 16S rRNA gene (*3*). The American Type Culture Collection (ATCC) 6940 *C. striatum* strain was included as phenotypic and molecular control. All strains were stored at –80°C until use.

MICs were determined by using microdilution in cation-adjusted Mueller-Hinton broth in accordance with guidelines of the Clinical and Laboratory Standards Institute (CLSI) (*4*). The following antimicrobial drugs were tested: tigecycline and piperacillin/tazobactam, oxacillin, gentamicin, kanamycin, levofloxacin, erythromycin, clindamycin, piperacillin, vancomycin, teicoplanin, tetracycline, moxifloxacin, imipenem, meropenem, quinupristin/dalfopristin, linezolid, and daptomycin. Etest strips (AB-BIODISK, Solna, Sweden) were used for vancomycin, teicoplanin, linezolid, and daptomycin. Daptomycin Etests were performed by using Muller-Hinton agar (Oxoid, Milan, Italy), supplemented to a final concentration of 50 mg/L calcium.

In the absence of approved breakpoints for *Corynebacterium* spp., we used those for α-hemolytic streptococci of the *viridans* group. Results were read after incubation at 37°C for 18–24 h. Susceptibility to daptomycin was defined as MIC <1 mg/L (*5*); CLSI guideline MIC breakpoints were used for all other drugs tested (*4*).

To further characterize the *C. striatum* isolates, we used 2 DNA fingerprinting techniques: automated ribotyping (RiboPrinter Microbial Characterization System; DuPont Qualicon, Wilmington, DE, USA) with *Eco*RI as restriction enzyme and pulsed-field gel electrophoresis (PFGE) macrorestriction analysis with 2 enzymes (*Xba*I and *Swa*I; New England Biolabs, Beverly, MA, USA). We had used 4 enzymes (*Xba*I, *Swa*I, *Sfi*I, and *Pac*I) to test 10 random strains, but because *Xba*I and *Swa*I enzyme-restriction patterns gave a better resolution for low and high molecular weight fragments, respectively, we used only these 2 restriction enzymes to type all 36 strains.

Whole genomic DNA chromosomal extraction, macrorestriction digestion, and PFGE (CHEF-DR II apparatus; Bio-Rad, Hercules, CA, USA) were performed as previously reported (*6*). Macrorestriction fragments were separated on 1% (wt/vol) ultrapure agarose gels (Sigma Aldrich, St. Louis, MO, USA) at 6 V/cm, for 21 h at 14°C with pulse times of 0.1–5 s, to separate *Xba*I fragments, and for 23 h with pulse times of 1–70 s, to separate *Swa*I fragments. Lambda DNA concatemers (New England BioLabs) were used as molecular size markers. Similarities among macrorestriction patterns were identified according to established criteria (*7*).

The sequence of pTP10 (GenBank accession no. AF024666) (*8*) was used to design the primers for *erm*(X), *tet*A and *tet*B, *cmx*, *aph*A1, and *rep*B genes. The VectorNTI program (Invitrogen, www.invitrogen.com) was used for this purpose. The presence of pTP10 was confirmed first by amplification and sequencing of the resistance determinants and the replication gene (*rep*B) and then by *Xba*I and *Swa*I PFGE hybridizations, performed with the specific probes (*erm*(X)*, tet*AB*, cmx*, and *aph*A1), following a protocol previously described (*9*). The PCR amplifications were performed in a Techne TC412 thermal cycler (Barloworld Scientific, Staffordshire, UK). All primers and the related probe regions used in hybridization experiments are shown in Table 1.

**Table 1:** Primer conditions, PCR products, and related sequences confirmed by BLAST analysis of 36 strains of multidrug-resistant *Corynebacterium striatum,* Italy, 2005–2007*

Primer	Related resistance	Sequence (5′ → 3′)	Temperature, °C	Size, bp	BLAST from–to, bp
*erm*X up *erm*X down	Erythromycin and clindamycin	AACCATGATTGTGTTTCTGAACG ACCAGGAAGCGGTGCCCT	57	566	2,285–2,850
*te*tA up *tet*B down	Tetracycline, oxytetracycline, and oxacillin	TTAGCGTTCGGCGACCTGG AACTGGGTGCCTTCAGGGTC	58	1,829	5,496–7,324
*cmx*B up *cmx*A down	Cloramphenicol (2 identical subunits)	AGTCGGTATGGTCGTCGGC GCTCCGATATTCAATGCTGCG	57	879	16,031–16,909 36,078–36,956
*aph*A1 up *aph*A1 down	Aminoglycoside	GGCAAGATCCTGGTATCGGTCT AGACTAAACTGGCTGACGGCAT	57	480	41,859–42,338
*rep*B up *rep*B down	Replicase	CGATCTGGAATTTGTCTGCCGT CTGGTTGATAGACCCCGTGT	57	875	32,523–33,397

*BLAST (http://blast.ncbi.nlm.nih.gov/Blast.cgi) analysis of each gene with pTP10 sequence (GenBank accession no. AF024666) showed nucleotide identities >99%.

All *C. striatum* isolates were recovered from hospitalized patients who had undergone surgery or been admitted to intensive care units (Table 2). We documented 19 cases of infections and discarded 17 as contaminants. The isolates that were considered causes of infections were responsible for 8 cases of ventilator-associated pneumonia (including 1 with associated pleural empyema), 2 cases of pneumonia, 1 case of catheter-related sepsis, 2 cases of ventilator-associated tracheobronchitis, and 6 cases of wound infections.

**Table 2:** Clinical diagnoses for 36 patients with *Corynebacterium striatum* infection, Italy, 2005–2007*

Specimens	No. isolates			Diagnosis
	Total	From ICU	From non-ICU wards	
BAL fluid, pleural fluid, blood, tracheal aspirate	8	7	1	Ventilator-associated pneumonia
BAL fluid	2	2	0	Ventilator-associated tracheobronchitis
BAL fluid, lung biopsy	2	0	2	Pneumonia
Blood, CVC tip	1	1	0	CVC-related bacteremia
CVC tip	1	1	0	CVC exit-site cellulites
Blood, surgical wound	5	1	4	Sternal wound cellulites and infections
Tracheal aspirate	10	10	0	Ventilator-associated respiratory tract colonization
CVC tip	6	4	2	CVC-exit site colonization
Urine	1	0	1	Urinary tract catheter colonization
Total	36	26	10	

*ICU, intensive care unit; BAL, bronchoalveolar lavage; CVC, central venous catheter.

The 36 strains showed an MDR phenotype, including resistance to >3 classes of drugs; MICs required to inhibit growth of 90% (MIC_90_) were penicillins >256 mg/L, carbapenems >256 mg/L, gentamicin 32 mg/L, levofloxacin 256 mg/L, tetracycline >256 mg/L, lincosamides >256 mg/L, and erythromycin 32 mg/L. *C. striatum* strains were susceptible to only the most recent drugs used for treatment of infections with gram-positive organisms, such as glycopeptides and tigecycline (MIC_90_ 1 mg/L), quinupristin/dalfopristin and daptomycin (MIC_90_ 0.25 mg/L), and linezolid (MIC_90_ 2 mg/L). A discrepancy was found when susceptibility testing using a disk-diffusion method was performed on different strains; the inhibition zone of erythromycin was always in the intermediate range, even if MICs for this drug were in the low-resistance range.

 Ribotyping gave a unique profile for all strains in this study. PFGE enabled us to discriminate the right number of macrorestriction fragments (*5*,*10*,*11*) for pattern comparison.

Analyses of *Swa*I digestion patterns showed that of the 36 strains, only 1 clone had 3 different subtypes (30 strains subtype a1, 4 strains a2, and 2 strains a3). Macrorestriction analysis with *Xba*I showed almost comparable results (27 strains A1, 7 strains A2, and 2 strains A3) (Figure). This genotyping method and the enzymes used were defined as appropriate, comparing PFGE patterns of our clinical isolates with *C. striatum* ATCC 6940 type strain, which was different with respect to the epidemic strains. This result demonstrates that single MDR *C. striatum* clones had been selected and were circulating in the 3 hospitals.

**Figure:**
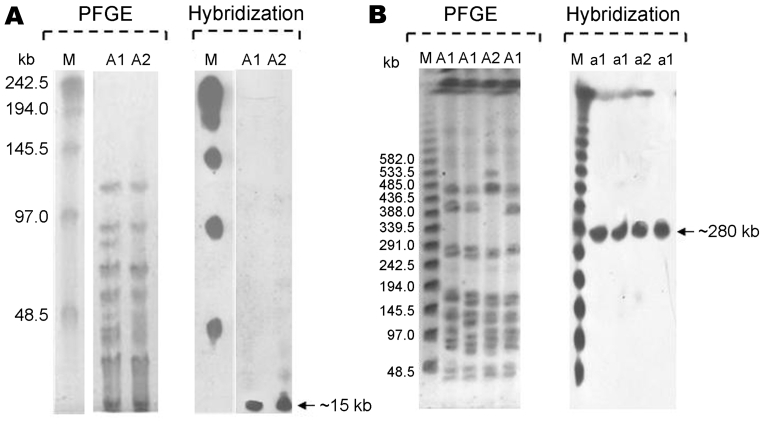
Pulsed-field gel electrophoresis (PFGE) patterns of *Corynebacterium striatum* and their representative hybridizations obtained with probes corresponding to the resistance genes *erm*(X)*, tet*A*-tet*B*, cmx*, and *aph*A1 (m, lambda ladder PFG marker). A) *Xba*I (A1and A2 profiles); B) *Swa*I (a1 and a2 profiles).

Further, the molecular characterization of some of the resistance genes in the 36 *C. striatum* isolates demonstrated the presence of *erm*(X), codifying for the resistance to erythromycin and clindamycin; *tet*A, and *tet*B, codifying for the resistance to tetracycline, oxytetracycline, and oxacillin; and *cmx* and *aphA*1, responsible for resistance to aminoglycosides and chloramphenicol, respectively. The presence of pTP10 carrying all these determinants was confirmed by amplification and sequencing of these genes and the replication gene of the plasmid, together with hybridization experiments demonstrating that all resistance determinants were localized in the same hybridization band generated by each probe onto PFGE*_XbaI_* (≈15 kb) and PFGE*_SwaI_* (≈280 kb) membranes (Figure).

## Conclusions

We report isolation of MDR *C. striatum* from clinical specimens responsible for cases of pneumonia, catheter-related bacteremia, and wound infections. Infections sustained from this species are strongly associated with devices, not only tubes or catheters (91%) but also sternal surgical wound wires.

The MDR phenotype of these strains was immediately observed and was responsible for the alarm that led to the subsequent in-depth examination of these strains. Their clonal nature, as demonstrated in our study, is of particular concern. Further, the MDR phenotype correlated to the presence of the pTP10 plasmid, which demonstrates that these MDR microorganisms acquired not only the capability to cause infections but also increased resistance and the ability to spread by virtue of their clonal nature. The only drugs still active against these MDR strains are glycopeptides, linezolid, quinopristin/dalfopristin, daptomycin, and tigecycline. To avoid using drugs that appear active in vitro but that could be ineffective in vivo, clinicians should be aware of the circulation of these MDR strains.

## References

[1] Mayall C, ed. Surveillance of nosocomial infections. 3rd ed. Philadelphia: Lippincott Williams & Wilkins; 2004.

[2] Renom F, Garau M, Rubi M, Ramis F, Galmes A, Soriano JB. Nosocomial outbreak of *Corynebacterium striatum* infection in patients with chronic obstructive pulmonary disease. J Clin Microbiol. 2007;45:2064–7. 10.1128/JCM.00152-07 17409213 PMC1933039

[3] Pascual C, Lawson PA, Farrow JA, Gimenez MN, Collins MD. Phylogenetic analysis of the genus *Corynebacterium* based on 16S rRNA gene sequences. Int J Syst Bacteriol. 1995;45:724–8. 7547291 10.1099/00207713-45-4-724

[4] Clinical and Laboratory Standard Institute. Performance standards for antimicrobial testing. Approved standards. Wayne (PA): The Institute; 2006.

[5] Iaria C, Stassi G, Costa GB, Biondo C, Gerace E, Noto A, Outbreak of multi-resistant *Corynebacterium striatum* infection in an Italian general intensive care unit. J Hosp Infect. 2007;67:102–4. 10.1016/j.jhin.2007.07.002 17719684

[6] Sampaio JLM, Chimara E, Ferrazoli L, da Silva Telles MA, Del Guercio VM, Jerico ZVN, Application of four molecular typing methods for analysis of *Mycobacterium fortuitum* group strains causing post-mammaplasty infections. Clin Microbiol Infect. 2006;12:142–9. 10.1111/j.1469-0691.2005.01312.x 16441452

[7] Tenover FC, Arbeit RD, Goering RV, Mickelsen PA, Murray BE, Persing DH, Interpreting chromosomal DNA restriction patterns produced by pulsed-field gel electrophoresis: criteria for bacterial strain typing. J Clin Microbiol. 1995;33:2233–9. 7494007 10.1128/jcm.33.9.2233-2239.1995 PMC228385

[8] Tauch A, Krieft S, Kalinowski J, Puhler A. The 51,409-bp R-plasmid pTP10 from the multiresistant clinical isolate *Corynebacterium striatum* M82B is composed of DNA segments initially identified in soil bacteria and in plant, animal, and human pathogens. Mol Gen Genet. 2000;263:1–11. 10.1007/PL00008668 10732668

[9] Mato R, Camapnile F, Stefani S, Crisostomo MI, Santagati M, Sanches SI, Clonal types and multidrug resistance patterns of methicillin-resistant *Staphylococcus aureus* (MRSA) recovered in Italy during the 1990s. Microb Drug Resist. 2004;10:106–13. 10.1089/1076629041310109 15256025

[10] Tarr PE, Stock F, Cooke RH, Fedorko DP, Lucey DR. Multidrug-resistant *Corynebacterium striatum* pneumonia in a heart transplant recipient. Transpl Infect Dis. 2003;5:53–8. 12791076 10.1034/j.1399-3062.2003.00002.x

[11] Martin MC, Melon O, Celada MM, Alvarez J, Mendez FJ, Vazquez F. Septicaemia due to *Corynebacterium striatum*: molecular confirmation of entry via the skin. J Med Microbiol. 2003;52:599–602. 10.1099/jmm.0.05102-0 12808083

